# Development and Examination of the Reactive Attachment Disorder and Disinhibited Social Engagement Disorder Assessment Interview

**DOI:** 10.1177/1073191118797422

**Published:** 2018-09-02

**Authors:** Stine Lehmann, Sebastien Monette, Helen Egger, Kyrre Breivik, David Young, Claire Davidson, Helen Minnis

**Affiliations:** 1 Regional Centre for Child and Youth Mental Health and Child Welfare -West, Uni Research Health, Bergen, Norway; 2 Department of Psychology, Université du Québec à Montréal (UQAM), Quebec, Canada; 3 Department of Child and Adolescent Psychiatry, New York University School of Medicine, NY, USA; 4 Department of Mathematics and Statistics, University of Strathclyde, NHS, Greater Glasgow and Clyde, Glasgow, Scotland, UK; 5 Adverse Childhood Experiences Clinical and Research Centre, Institute of Health and Wellbeing, University of Glasgow, Glasgow, Scotland, UK

**Keywords:** RAD, DSED, CFA, assessment, adolescence, attachment disorder

## Abstract

The fifth edition of the *Diagnostic and Statistical Manual* (*DSM*) categorizes reactive attachment disorder (RAD) and disinhibited social engagement disorder (DSED) as two separate disorders, and their criteria are revised. For DSED, the core symptoms focus on abnormal social disinhibition, and symptoms regarding lack of selective attachment have been removed. The core symptoms of RAD are the absence of attachment behaviors and emotional dysregulation. In this study, an international team of researchers modified the Child and Adolescent Psychiatric Assessment for RAD to update it from *DSM-IV* to *DSM-5* criteria for RAD and DSED. We renamed the interview the reactive attachment disorder and disinhibited social engagement disorder assessment (RADA). Foster parents of 320 young people aged 11 to 17 years completed the RADA online. Confirmatory factor analysis of RADA items identified good fit for a three-factor model, with one factor comprising DSED items (indiscriminate behaviors with strangers) and two factors comprising RAD items (RAD1: failure to seek/accept comfort, and RAD2: withdrawal/hypervigilance). The three factors showed differential associations with clinical symptoms of emotional and social impairment. Time in foster care was not associated with scores on RAD1, RAD2, or DSED. Higher age was associated with lower scores on DSED, and higher scores on RAD1.

## Background

The construct of attachment disorder links early maltreatment to later psychopathology (Goldfarb, 1945a, 1945b; Tizard & Rees, 1975). An attachment disorder is defined as “markedly disturbed and developmentally inappropriate social relatedness in most social contexts” (Rutter, Kreppner, & Sonuga-Barke, 2009, p. 535), presenting before the age of 5 years, and originating from very depriving and pathogenic care conditions. In *DSM-IV*, attachment disorder was assumed to be one disorder with two subtypes: disinhibited reactive attachment disorder ([RAD]; socially indiscriminate behavior) and inhibited RAD (lack of comfort seeking and withdrawal; Zeanah & Gleason, 2015). These were assumed to share the etiology of exposure to physical and social neglect and abuse and an absence of adequate caregiving during childhood (*DSM-IV*; American Psychiatric Association, 1994).

In the fifth edition of the *DSM* (*DSM-5*; American Psychiatric Association, 2013), the construct of attachment disorders was revised. The cluster of symptoms relating to indiscriminate behaviors is now regarded as a disorder called disinhibited social engagement disorder (DSED), which is related to, but *separate from*, RAD. RAD now refers to the cluster of inhibited symptoms only. Both RAD and DSED are categorized under the chapter “Trauma- and Stressor-Related Disorders” in *DSM-5* and are still considered associated with severe pathogenic care.

The main empirical support for DSED and RAD as discrete constructs of child psychopathology originates from two longitudinal studies on children raised in extremely deprived institutional contexts: the English and Romanian Adoptees Study (O’Connor, Bredenkamp, Rutter, & English and Romanian Adoptees Study Team, 1999; Sonuga-Barke et al., 2017) and the Bucharest Early Intervention Project (Smyke, Dumitrescu, & Zeanah, 2002; Zeanah, Humphreys, Fox, & Nelson, 2017). RAD and DSED have predictable associations with risk factors (including attachment), cause functional impairment, and, particularly in the case of DSED, can persist over time (Gleason et al., 2011). The results of these seminal studies have heavily influenced the reconceptualization of attachment disorder in *DSM-5* (Zeanah & Gleason, 2010; 2015).

*DSM-IV* (American Psychiatric Association, 1994) defined the disinhibited subtype of RAD (now known as DSED) as “evidenced by diffuse attachments as manifest by indiscriminate sociability with marked inability to exhibit appropriate selective attachments” (p. 118). The new *DSM-5* diagnostic criteria for DSED comprises two criteria: A and B. According to Criterion A, the child must exhibit at least two of the following symptoms: lack of reticence around unfamiliar adults; being too physically or verbally close; not checking back with caregiver in unfamiliar setting; and/or willingness to go off with an unfamiliar adult. Criterion B states that the disinhibited behavior is not limited to impulsivity but includes social disinhibition. Symptoms relating to a lack of selective attachment (e.g., “diffuse attachment,” “inability to exhibit appropriate selective attachments,” “lack of selectivity in choice of attachment figures”) were removed, demonstrating that DSED is regarded almost exclusively as a disorder of social relatedness and not of attachment.

The inhibited subtype of RAD in *DSM-IV* (American Psychiatric Association, 1994) was defined as “evidenced by a persistent failure to initiate or respond in a developmentally appropriate fashion to most social interactions, as manifest by excessively inhibited, hyper vigilant, or highly ambivalent and contradictory responses” (p. 118). The *DSM-5* diagnostic criteria for RAD comprise Criteria A and B. Criterion A comprises both minimal comfort seeking and minimal responding to comfort. Criterion B requires at least two of the following symptoms: minimal social/emotional responsiveness; limited positive affect; and/or unexplained or sudden irritability/sadness/fearfulness. In *DSM-5*, symptoms overlapping with behaviors suggestive of disorganized attachment (e.g., “highly ambivalent and contradictory responses”) have been removed to focus on the absence of attachment behavior described in Criterion A. In addition, Criterion B describes social and emotional disturbances, closely related to relational trauma reactions. Behaviors suggestive of posttraumatic stress disorder (e.g., “hyper vigilance,” “may exhibit frozen watchfulness”) have been replaced by more general emotional dysregulation criterion (unexplained or sudden irritability/sadness/fearfulness (Criterion B3).

The purpose of the *DSM-5* separation of Criterion A (lack of attachment behavior) and Criterion B (social/emotional disturbances) was to restrict the diagnosis of RAD to individuals in whom both disturbances are present (Zeanah & Gleason, 2010). As the vast majority of empirical studies on RAD and DSED stem from samples of institutionalized children, caution is warranted in generalizing findings from studies of institutionalized children with limited access to stable attachment figures to maltreated children raised in a family context (Glowinski, 2011). The quality of care in institutions may differ from the characteristics of care in a dysfunctional family, where the carer is not necessarily physically absent. Furthermore, family-raised children have often been exposed to maltreatment from their primary attachment figure. For maltreated children raised in a family context, this separation of Criteria A and B may be central: While the child may have an attachment figure and exhibit attachment behavior, behavior compliant with Criterion B may also be exhibited due to exposure to relational trauma. Hence, for noninstitutionalized children, Criterion A and Criterion B may constitute two separate constructs. In line with this, one might expect that symptoms defining Criterion B have a higher overlap with other symptoms of emotional and relational disturbances, and less so with symptoms defining Criterion A.

### Existing Measures of RAD and DSED Symptoms

In the following section, we provide a brief overview of measures for assessing RAD and DSED. A complete overview of available measures including references are presented in Table 1.

**Table 1.** Existing Measures of Reactive Attachment Disorder and Disinhibited Social Engagement Disorder.

Characteristics of the measure		Validation studies
*Semistructured interview*		
CAPA-RAD (Child and Adolescent Psychiatric Assessment–RAD Module)		
Original version	Minnis et al. (2009)	Davidson et al. (2015): DV
Current version	Minnis et al. (2009)	Follan et al. (2011): IC, IRA, DV
RAD:DSED item ratio	4:6	Kay, Green, and Sharma (2016): IC, CV
Age range:	S	Minnis et al. (2009): IRA, CV
Nosological classification	*DSM-IV* and ICD-10	Minnis et al. (2013): CV
CSRCM (Caregiver Selective Relationship Composite Measure)		
Original version	Roy, Rutter, and Pickles (2004)	Roy et al. (2004): IRA, CV
Current version	Roy et al. (2004)	
RAD:DSED item ratio	3:1	
Age range	S	
Nosological classification	none	
DAI (Disturbance of Attachment Interview)		
Original version	Smyke and Zeanah (1999)	Gleason et al. (2011): CV, DV
Current version	Zeanah, Smyke, Koga, Carlson, and the BEIP Group (2005)	Humphrey, Nelson, Fox, and Zeanah (2017): IC, CV
RAD:DSED item ratio	5:3	Giltaij, Sterkenburg, and Schuengel (2017): IRA, CV
Age range	P, S, A	Jonkman et al. (2014): IRA, CV
Nosological classification	*DSM-IV* alternative (Boris, Zeanah, Larrieu, Scheeringa, & Heller, 1998)	Oliveira et al. (2012): IRA, CV
		Oosterman and Schuengel (2007): FV, IC
		Smyke and Zeanah (1999): IC, IRA
		Soares et al. (2014): IC
		Vervoort, De Schipper, Bosmans, and Verschueren (2013): IC, FV, CV, DV
		Zeanah et al. (2005): CV
		Zeanah, Smyke, and Dumitrescu (2002): IC, CV
DASSI (Disinhibited Attachment Semi-Structured Interview)		
Original version	O’Connor et al. (1999)	Bruce, Tarullo, and Gunnar (2009): IRA, IC, CV
Current version	Rutter et al. (2007)	Garvin, Tarullo, Ryzin, and Gunnar (2012): IRA, DV
RAD:DSED item ratio	0:3	O’Connor et al. (1999): IC, DV
Age range	P, S	O’Connor and Rutter (2000): IRA, IC, DV
Nosological classification	None	O’Connor, Marvin, Rutter, Olrick, and Britner (2003): IRA, IC, CV
		Rutter et al. (2007): IC, IRA, CV, DV
		Rutter, Kreppner, and O’Connor (2001): DV
		Zeanah et al. (2002): IC, CV
DSED Interview		
Original version	Lawler, Koss, Doyle, and Gunnar (2016)	Lawler et al. (2016): IC, IRA, CV
Current version		
RAD:DSED item ratio	0:4	
Age range	P	
Nosological classification	Near *DSM-5* (3/4 DSED criteria)	
PAPA (Preschool Age Psychiatric Assessment) RAD section		
Original version	Egger, Ascher, and Angold (1999)	Gleason et al. (2011): CV
Current version	Egger et al. (1999)	
RAD:DSED item ratio	12:4	
Age range	I, P	
Nosological classification	*DSM-IV* and ICD-10	
RADA (RAD and DSED Assemment)		
Original version	Lehmann et al. (manuscript submitted)	Lehmann et al. (manuscript submitted): FV
Current version	Lehmann et al. (manuscript submitted)	Archambault et al. (Submitted): IC, FV, CV
RAD:DSED item ratio	11:9	
Age range	S, A	
Nosological classification	*DSM-5*	
5IF (Five-Item Indiscriminately Friendliness Behavior measure)		
Original version	Chisholm, Carter, Ames, and Morison (1995)	Chisholm et al. (1995): DV
Current version	Chisholm et al. (1995)	Chisholm (1998): IRA, DV
RAD:DSED item ratio	0:5	Dobrova-Krol, Bakermans-Kranenburg, Van Ijzendoorn, & Juffer (2010): IC, DV
Age range	P, S	McCall et al. (2016): IC
Nosological classification	None	Pears, Bruce, Fisher, and Kim (2010): IC, TRT
		Van den Dries, Juffer, Van Ijzendoorn, Bakermans-Kranenburg, and Alink (2012): IRA, TRT, DV
		Zeanah et al. (2002): IC, CV
*Questionnaire (completed by caregiver)*		
ETRADD-Q (Early Trauma Related and Dysregulation Disorders Questionnaire) Short Version		
Original version	Monette (2016)	Monette, Cyr, Terradas, Couture, and Archambault (2017): IC, FV, CV
Current version	Monette (2016)	
RAD:DSED item ratio	8:8	
Age range	S	
Nosological classification	*DSM-5*	
DAWBA-RAD (Development and Wellbeing Assessment–RAD Section)		
Original version	Minnis and Goodman (n.d.)	Lehmann, Breivik, Heiervang, Havik, and Havik (2016): FV, DV
Current version	Lehmann et al. (2016)	Kay and Green (2013): FV
RAD:DSED item ratio	5:9	Kay and Green (2016): IC
Age range	S	
Nosological classification	*DSM-IV* and ICD-10	
RPQ (Relationship Problem Questionnaire)		
Original version	Minnis, Rabe-Hesketh, and Wolkind (2002)	Doku (2016): DV
Current version	Vervoort et al. (2013)	Kay et al. (2016): IC, CV
RAD:DSED item ratio	6:4	Millward, Kennedy, Towlson, and Minnis (2006): DV
Age range	S	Minnis et al. (2013): CV
Nosological classification	*DSM-IV* and ICD-10	Minnis et al. (2009): IC, CV
		Minnis et al. (2007): IC, FV, DV
		Minnis et al. (2002): IC, TRT, CV, FV
		Monette et al. (2017): CV, IC
		Spilt et al. (2016): DV
		Vervoort et al. (2013): FV, IC, CV, DV
		Vervoort, Bosmans, Doumen, Minnis, and Verschueren (2014): CV, DV
*Structured observation*		
AFRS (Attachment Formation Rating Scale)		
Original version	Carlson (2002/2011)	Carlson, Hostinar, Mliner, and Gunnar (2014): IRA, TRT, DV
Current version	Carlson (2002/2011)	Dobrova-Krol et al. (2010): IRA
RAD:DSED item ratio	1:0	Gleason et al. (2014): DV
Age range	I, P	Zeanah et al. (2005): IRA, CV
Nosological classification	None	
DSA (Disinhibited Social Approach)		
Original version	Lawler, Hostinar, Mliner, and Gunnar (2014)	Lawler et al. (2014): IRA
Current version	Lawler et al. (2014)	Lawler et al. (2016): VC
RAD:DSED item ratio	0:14	
Age range	I, P	
Nosological classification	None	
DSBOM (Disinhibited Social Behavior Observational Measure)		
Original version	Bruce et al. (2009)	Bruce et al. (2009): IRA, IC, CV
Current version	Tarullo, Garvin, and Gunnar (2011)	Tarullo et al. (2011): IRA
RAD:DSED item ratio	0:3	
Age range	P	
Nosological classification:	None	
Investigator rating of physical contact (age 6)		
Original version	Rutter et al. (2007)	Rutter et al. (2007): IRA, CV
Current version	Rutter et al. (2007)	
RAD:DSED item ratio	0:1	
Age range	P	
Nosological classification	None	
Investigator rating of children’s interaction (age 11)		
Original version	Rutter et al. (2007)	Kay et al. (2016): IC, CV
Current version	Rutter et al. (2007)	Rutter et al. (2007): IC, IRA, FV, CV
RAD:DSED item ratio	0:8	
Age range	S	
Nosological classification	None	
OSR (Observation Schedule for RAD)		
Original version	Minnis et al. (2009)	Davidson et al. (2015): DV
Current version	McLaughlin, Espie, and Minnis (2010)	Follan et al. (2011): CV, DV
RAD:DSED item ratio	0:10	McLaughlin et al. (2010): IC, CV
Age range	S	Minnis et al. (2009): IRA, CV
Nosological classification	*DSM-IV* and ICD-10	Vervoort et al. (2013): FV, IC, CV
RISE (Rating for Infant–Stranger Engagement)		
Original version	Riley, Atlas-Corbett, and Lyons-Ruth (2005)	Lalande et al. (2014): IRA, DV
Current version	Riley et al. (2005)	Lyons-Ruth, Bureau, Riley, and Atlas-Corbett (2009): IRA, TRT, DV
RAD:DSED item ratio	0:1	Oliveira et al. (2012): IRA, DV, CV
Age range	I, P	
Nosological classification	None	
StrD procedure (Stranger at the Door procedure)		
Original version	Gleason et al. (2014)	Gleason et al. (2011): IRA, CV
Current version	Gleason et al. (2014)	Gleason et al. (2014): DV, CV
RAD:DSED item ratio	0:1	
Age range	P	
Nosological classification	None	

*Note.* RAD = reactive attachment disorder; DSED = disinhibited social engagement disorder; IC = reliability (internal coherence); IRA = reliability (interrater agreement); TRT = reliability (test–retest); FV = factorial validity; CV = convergent validity; DV = divergent validity; I = infant, P = preschooler, S = school-age children, A = adolescents; ICD-10 = the international classification of diseases, tenth revision (World Health Organization, 1992).

#### Structured Observation Instruments

Two structured observational instruments are based on the administration of the Strange Situation Procedure (SSP; Ainsworth & Bell, 1970): The Attachment Formation Rating Scale (Zeanah et al., 2005) and the Rating for Inhibited Attachment Behavior (Corval, Belsky, Baptista, Mesquita, & Soares, 2018) for evaluating RAD symptoms. The Rating for Infant–Stranger Engagement (Lyons-Ruth et al., 2009) assesses disinhibited behavior, again during the SSP. Other observational instruments such as the Disinhibited Social Behavior Observational Measure (Bruce et al., 2009) involve videotaped laboratory interaction between a child and an adult stranger who gradually initiates contact with the child. The Observation Schedule for RAD (Minnis et al., 2009) codes child behavior in a clinic waiting room in the presence of a stranger. Finally, The Stranger at the Door procedure (Gleason et al., 2011) is a simulated situation whereby an assessor, who is a stranger to the child, knocks on the door of the child’s home and invites the child to go off with him or her. Most instruments focus on DSED symptoms only, and none assess DSED and RAD symptoms simultaneously.

#### Screening Questionnaires

The 10-item standardized screening tool, the Relationship Problems Questionnaire (RPQ) was developed and validated with noninstitutionalized samples of children in foster care (Millward et al., 2006; Minnis et al., 2002), and it has been used successfully to identify RAD and DSED symptoms in large general population studies (Minnis et al., 2007), and in clinical samples (Vervoort et al., 2013) of school-aged children. Population norms are not yet available for a new 11-item version. A second newly developed instrument, the Early TRAuma-related Disorders Questionnaire–Short Version (ETRAD-Q-SV; Archambault, Monette, Cyr, Terradas, & Couture, 2017) is a 16-item screening tool for RAD and DSED based on *DSM-5* criteria. A longer version is presently undergoing validation. Diagnostic assessment requires more comprehensive tools, which assess not only symptoms but also their impact on everyday functioning.

#### Semistructured Interviews

The Five-Item Indiscriminately Friendly Behavior (Chisholm et al., 1995) was one of the first tools developed to assess DSED. Although the Five-Item Indiscriminately Friendly Behavior is not based on the *DSM-5*, the items measured relate to the four core criteria required in *DSM-5*. The Disinhibited Attachment Semi-Structured Interview (O’Connor et al., 1999; Rutter et al., 2007) was used primarily by the English and Romanian Adoptees Study team at a time when practically no other measures of RAD/DSED existed. The psychometric properties reported are acceptable, although factor analysis is not possible as the measure consists of only three items. The Disturbance of Attachment Interview developed by the Bucharest Early Intervention Group (Smyke et al., 2002) comprises five items measuring RAD symptoms and three items assessing DSED symptoms. This interview has identified RAD and DSED symptoms in noninstitutionalized maltreated preschool foster children (Jonkman et al., 2014; Oosterman & Schuengel, 2007; Zeanah et al., 2004). The interview shows a two-factor structure, good internal consistency, good interrater agreement, and good convergent and divergent validity indices. Although the measure assesses both RAD and DSED, the tool only partly fulfills the *DSM-5* criteria: For DSED, Criterion A2 (being too physically or verbally close) is not covered, and for RAD, Criterion B2 (limited positive affect) is not covered.

The Child and Adolescent Psychiatric Assessment-RAD assessment (CAPA-RAD) is one module of a broader diagnostic interview (Angold et al., 1995). There are four items specific to RAD and six items specific to DSED and diagnostic classification is based on *DSM-IV* criteria (Minnis et al., 2013). In addition to core DSED and RAD items, items suggested by experts in child abuse and neglect as well as foster and adoptive carers were added. These items do not contribute to the diagnosis of RAD or DSED but are intended to contribute to the overall clinical formulation of the child psychological profile (Minnis et al., 2009). The CAPA-RAD has good interrater reliability, internal consistency, convergent validity, and good specificity, successfully distinguishing children with DSED from controls. The CAPA-RAD was later modified by Minnis and Goodman to be utilized as a RAD section within the diagnostic interview Developmental and Wellbeing Assessment (DAWBA; Goodman, Ford, Richards, Gatward, & Meltzer, 2000), originally comprising 24 items (Kay & Green, 2013) and later being reduced to 14 items (Lehmann, Havik, Havik, & Heiervang, 2013). The advantage of the DAWBA-RAD section, especially for large-scale research purposes, is that it may be completed online, through a secure website. However, it should be noted that the DAWBA must be administered as a whole; select modules such as the RAD module cannot be administered individually.

This brief review of existing instruments measuring RAD and DSED demonstrates that there are no fully validated instruments based on the updated criteria of the *DSM-5*. Furthermore, there are no structured observational instruments that enable assessment of RAD and DSED simultaneously. Existing observational instruments focus almost exclusively on DSED but do not entirely cover the *DSM-5* DSED symptoms. Many of these instruments are also hard to use in clinical settings due to the amount of administration time. Of the available semistructured interviews, the Disturbances of Attachment Interview, the CAPA-RAD, and the DAWBA-RAD stand out because of strong psychometric properties and joint measurement of both RAD and DSED. However, none of these instruments have yet been updated to meet the *DSM-5* criteria.

### Measuring RAD and DSED in Adolescence

During adolescence, the role of peers becomes more prominent, and a central developmental task is to become less dependent on primary attachment figures. This involves transference of dependencies from parental to peer relationships (Allen, 2008). The ability to get along with peers may be seen as one of several precursors for social and emotional well-being (Allen & Antonishak, 2008). The English and Romanian Adoptees Study and the Bucharest Early Intervention Project followed the development of the children from early childhood into adolescence, and therefore, the need for developmentally appropriate assessment methods arose. In the English and Romanian Adoptees Study follow-up of 11-year-old adoptees, Rutter et al. (2007) modified the Disinhibited Attachment Semi-Structured Interview to capture DSED symptoms in young people. This interview was administered with carers and was combined with observational data. Modifications reflected children’s shifting focus from primary attachment figures to quality of peer relationships. The quality of peer relations, as a proxy for attachment security, was also measured when the children were 11 years old, via the Rutter Parents and Teacher Scale, as opposed to the SSP when children were 4 and 6 years old. Assessments of peer relations did not particularly target indiscriminate behavior toward peers.

The Bucharest Early Intervention Project continued to use the semistructured Disturbances of Attachment Interview (Smyke et al., 2002) to assess 8-year-old children (Smyke et al., 2012) and at follow-up when they were 12 years old. Findings demonstrated that caregiving disruptions in early life continued to have an effect throughout development and manifested as disturbances of attachment and social behaviors in early adolescence (Humphreys et al., 2017).

Studies using standardized measures of RAD and DSED have also strengthened the evidence that, in noninstitutionalized toddlers, school-aged children, and adolescents, RAD and DSED are relevant descriptions of their maltreatment-associated disorders (Boris et al., 2004; Kay & Green, 2013; Kočovská et al., 2012; Lehmann et al., 2016; Millward et al., 2006; Minnis et al., 2002; Oosterman & Schuengel, 2007; Pears et al., 2010; Vervoort et al., 2013; Zeanah et al., 2004). Furthermore, RAD and DSED have been shown to persist throughout childhood and the latter even into early adulthood (Sonuga-Barke et al., 2017). This indicates that, as in infants, there is a need to assess symptoms of RAD and DSED when studying mental health in older children and adolescents subjected to maltreatment.

Nevertheless, these issues are still under debate. In their research review, Zeanah and Gleason (2015) call into question whether the instruments used to assess disordered attachment behavior in noninstitutionalized young people beyond early childhood actually measure a broader phenomenon than that defined by the *DSM-5*. There is therefore a need to further examine the methods and measures required in order to effectively study RAD and DSED as defined in *DSM-5*, especially in adolescence. This is the aim of the current study.

### Objectives

The first aim of the study was to update and modify the CAPA-RAD interview to (a) correspond to the *DSM-5* criteria for RAD and DSED and (b) enable the assessment of RAD and DSED symptoms in adolescents. The second aim was to examine the factor structure of this modified interview, with the use of confirmatory factor analyses (CFA). Based on the *DSM-5*, we tested two alternative models: a two-factor structure, with items measuring DSED behavior and items measuring RAD behavior comprising one overall factor each, and a three-factor structure, one factor being DSED and with RAD having two factors (Cluster A symptoms and Cluster B symptoms in *DSM-5*, respectively). Third, we explored the possible associations between the reactive attachment disorder and disinhibited social engagement disorder assessment (RADA) factors established by the CFA and the formulation items in the RADA. We also tested whether time in foster care and child age was associated with RAD and DSED symptoms, respectively.

## Method

### Procedure and Study Sample

The study sample is part of the ongoing research project “Young in Foster Care” within the larger project Children at Risk Evaluation (CARE) models. Data were collected between October 1, 2016, and March 31, 2017. Eligible foster youth were born between 1999 and 2005 and had lived in their current foster home for at least 6 months following legally mandated placement. All were placed by municipalities in the five counties encompassed by the Office for Children, Youth and Family Affairs–Region South. Participants were assessed for eligibility from regional records (*N* = 573) and from the 43 municipal child protection service (*N* = 279) in the same region. Head of office in the child protection service were asked to provide background information for all eligible youths; in total, 740 foster youth were identified as eligible.

Foster parents were invited by postal mail out to participate: An information letter describing the study and how to complete the questionnaires was enclosed, and the parents were invited to complete the questionnaire either online or via telephone interview. Both foster mothers and foster fathers were asked to complete the questionnaire. Reminders were sent by post, and subsequent telephone contact. Foster parents were not compensated for participating.

The RADA was completed by foster parents of 320 youths (43.2% response rate): 277 foster mothers and 43 foster fathers.

### Measures: Instrument Development

The lead researchers from each of the three participating countries (HM, Scotland; SM, Canada; SL, Norway) examined the items from the existing English, French, and Norwegian translations of the CAPA-RAD interview. The aim was to develop the same interview for all three languages. Iterative discussions were held to calibrate the interviews prior to any modifications. Items in the CAPA-RAD interview had previously been translated into Norwegian (SL) and French (SM) and then back-translated, both approved by HM. The Norwegian translation of the interview originated from the Preschool version, the PAPA RAD interview (Egger et al., 1999); therefore, it comprised somewhat different items than the English original CAPA-RAD (only some of the English items had originated from the PAPA). We therefore calibrated the Norwegian version with the English original version, with the agreement of all authors, to make sure that we had the same items in all versions before we started the modification of the English version.

#### Items Updated to *DSM-5* RAD Criteria

To comply with new and more clearly defined criteria for RAD in *DSM-5*, new items were added; in total, 9 of the 11 RAD items are new or somewhat modified, and 7 of them are modified versions of items derived from the preschool version (PAPA RAD).To give an example, the original item *Failure to seek or accept comfort* was separated into two items—*Inability to seek comfort* and *Inability to accept comfort*—to comply with *DSM-5* Criteria A1 and A2. Also, the original items *Social and emotional withdrawal* and *Avoids eye contact* were supplemented with an additional item, *Avoids physical contact*, to more fully cover Criterion B1. Two items, *Limited positive affect* and *Difficulties being affectionate*, were added to comply with Criterion B2. To cover Criterion B3, the original item *Hypervigilance* was kept, but two new items were added: *Approach/avoidance toward carers* and *Emotional unpredictability*. The latter is a reformulation of *Unpredictable reunion response*, as this addresses a wider spectrum of social responses toward the caregiver (e.g., anger/irritability, sadness, or fear for no apparent reason).

#### Items Updated to *DSM-5* DSED Criteria

Items assessing DSED are predominantly the same as in the original CAPA-RAD. Nine items comprise the DSED scale in the RADA (Table 2). Two new items, originating from the PAPA-RAD, were added: First, *Wandering off with a stranger* was included to comply with Criterion A4. Second, *Indiscriminate peer relationships* was included together with the original CAPA-RAD item, *Demanding/attention seeking*, to cover Criterion B.

**Table 2.** Response Frequencies of Items in the Reactive Attachment Disorder and Disinhibited Social Engagement Disorder Assessment Interview, Completed by Foster Parents (*N* = 320).

Item No.	*DSM-5* criteria		Response frequencies, %		
			No	A little	A lot
		*DSED items*			
1	A1	Indiscriminate adult relationship	70.0	20.6	9.4
2	A1	Cuddliness with strangers	84.4	12.8	2.8
3	A1	Comfort seeking with strangers	86.7	7.9	5.4
4	A2	Personal questions	73.1	20.3	6.6
5	A2	Invading social boundaries	74.8	16.0	9.1
6	A3	Minimal checking back	72.2	19.7	8.1
7	A4	Wandering off with a stranger	76.9	17.2	5.9
8	B	Indiscriminate peer relationships	69.7	18.1	12.2
9	B	Demanding/attention seeking	41.9	33.8	24.2
		*RAD items*			
10	A1	Inability to seek comfort	45.0	43.8	11.3
11	A2	Inability to accept comfort	52.2	42.8	5.0
12	B1	Emotional and social withdrawal	52.8	32.2	15.0
13	B1	Avoids eye contact	60.9	30.6	8.4
14	B1	Avoids physical contact	66.9	23.1	10.0
15	B2	Limited positive affect	39.1	29.4	31.6
16	B2	Difficulties being affectionate	48.1	38.1	13.8
17	B3	Emotional unpredictability	55.0	27.7	17.3
18	B3	Approach/avoidance to carers	59.4	30.5	10.1
19	B3	Hypervigilance	64.8	27.0	8.2
20	B3	Frozen watchfulness	86.2	8.8	5.0
		*Formulation items*			
21		Misunderstand emotion	49.4	33.3	17.3
22		Negative attitude toward self	55.7	34.6	9.7
23		Self-harm	88.3	11.4	.3
24		Lack of remorse	17.0	58.8	24.2
25		Lack of empathy	37.5	43.5	18.9
26		Need to be in control	36.8	30.5	32.7
27		False affection	60.9	29.0	10.1
28		Hanging on behavior	62.3	27.0	10.7
29		Possessiveness	65.6	26.2	8.2
30		Pseudo-adult behavior	52.4	30.9	16.7
31		Abnormal eating pattern: gorging	63.1	23.0	13.9
32		Abnormal eating pattern: stealing	83.6	12.3	4.1

*Note. DSM-5* = *Diagnostic and Statistical Manual* (5th ed.); DSED = disinhibited social engagement disorder; RAD = reactive attachment disorder.

#### Additional (Formulation) Items

In addition to the 20 items measuring core symptoms of either RAD (11 items) or DSED (9 items), we kept 12 “formulation” items from the original version of the CAPA-RAD. These are items that do not contribute to RAD/DSED diagnosis, but which may contribute to clinical formulation of the child’s psychosocial functioning. These items were added during the development of the original CAPA-RAD interview via consultation with adoptive parents, foster carers, and clinical expert s in abuse and neglect (Minnis et al., 2009, web appendix). In the present study, the degree of overlap between these items and the RAD/DSED factors are examined.

#### Modification of Items to Also Assess Adolescents

Each item in the original CAPA-RAD was examined for its applicability to adolescents by HM and SL. The following four items were amended: The DSED item *Minimal checking back* was reworded to assess young people who act too independent for their age; *Does she/he fail to let you know where s/he is, and/or when she/he is coming home?*; and *Cuddliness with strangers* was reworded to also include being too physically close with unfamiliar peers. The formulation items *Hanging on behavior* was reworded to include clinging behavior toward peers; and *Possessiveness* was reworded to include possessive behavior toward peers.

#### Item Reduction

From the original CAPA-RAD, six items were removed as they were too ambiguous. For example; *High intensity behavior* may refer to emotional intensity or suggest hyperactivity problems. Furthermore, it may be too difficult to distinguish *Failure to learn from mistakes* and *Immature behavior* from problems relating to developmental delay. An additional four items were deleted from the Norwegian version of the CAPA-RAD, because they were originating from the preschool version and were therefore not relevant to the age-group.

#### Cultural Adjustments

In Nordic countries, children seldom or never use surnames to address adults. Therefore, the original formulation item Pseudo-adult behavior (*Does she/he quickly get on first name terms with adults?*) was amended to ask if the child quickly interacts with the adult as if they were on equal footing. This to ensure relevance across Nordic and British child-rearing practices.

### The RADA

We renamed the modified interview the reactive attachment disorder and disinhibited social engagement disorder assessment (RADA). Items underwent a Norwegian SL)/French translation (SM) and back-translation, both approved by HM. The RADA is currently available in French, Norwegian, and English. The RADA may be administered as an online assessment completed by carers or be administered as a face-to-face structured interview with carers, using paper format. The online version is particularly suitable for large-scale research projects, where face-to face assessment may be too demanding.

#### Scoring Instructions

The symptoms should have been present for the past year and should be coded only if they have been noted within the past 3 months unless, for selected items, they are coded as having “ever” been present. Answers on each item are coded on a 3-point scale as No (= 0), A little (= 1), A lot (= 2), yielding a scale range of 0 to 22 for the RAD scale and 0 to 18 for the DSED scale. Where responders tick off either 1 or 2 on any of the 20 items, they are given an open-ended question asking them to give an example of the behavior. The RADA has five additional questions at the end of the questionnaire to assess impact and social burden of the behavior (Does this worry you? Has she/he always been like that? Does this affect how well she/he gets along with the family and his/her ability to build and keep friendship? and Does this behavior put him/her in danger). These are scored on a 3-point scale: No (= 0), A little (= 1), A lot (= 2). The impact scale ranges from 0 to 10.

### Ethics

The Regional Committee for Medical and Health Research Ethics, Western Norway, approved the study. The Norwegian Directorate for Children, Youth and Family Affairs provided exemptions from confidentiality for caseworkers and foster parents. In accordance with Norwegian Ethics requirement, oral assent is required from children aged 12 years or older. The youths were instructed in their invitation letters that they could inform their foster parents if they did not want their foster parents to participate in the study.

### Statistical Analyses

Frequency distributions were analyzed with the IBM SPSS Statistics for Windows, Version 25. Mean scale scores were computed by dividing the sum score of each scale by the number of items in the scale. CFA was performed using the Lavaan package in R (Rossel, 2012). The models were examined using data from the 320 online interviews completed by foster parents of youths aged 11 to 17 years. The CFA models were estimated using a robust diagonally weighted least squares estimator (DWLS) with DELTA parameterization, to account for the multivariate nonnormality and the categorical data (ordinal data with three options; Dumenci & Achenbach, 2008; Flora & Curran, 2004).

First, a two-factor model corresponding to the *DSM-5* definition of RAD and DSED as two separate disorders was tested. Second, we tested an alternative model comprising three factors, corresponding to the *DSM-5* subcategorization of DSED and RAD as two clusters; RAD1, a pattern of inhibited, emotionally withdrawn behavior; and RAD2, social and emotional disturbances. For empirical identification of the three-factor model, an equality constraint had to be imposed on the unstandardized factor loadings of the two indicators measuring RAD1 (Kline, 2016). The fit of the CFA models was evaluated according to standard fit indices (Jackson, Gillaspy, & Purc-Stephenson, 2009). The recommended cutoffs for adequate fit are confirmatory fit index [CFI] ⩾ .90 and root mean square error of approximation [RMSEA] < .08, when using the DWLS estimator (Brown, 2006; Yu & Muthen, 2002). Tucker-Lewis index (TLI) of .95 or greater indicate a good model fit (Hu & Bentler, 1999).

In estimating reliability of the three new subscales in the RADA, we used the omega alpha coefficient (ω), as described in McDonald (1978). We employed the procedure described by Stone et al. (2013), and calculated the reliability of each factor in the final model using the formula from Green and Yang (2009), as implemented in the R package SemTools 0.4-14.

Correlation analyses with latent variables and DWLS as estimator were conducted where (a) DSED, RAD1, and RAD2 were correlated with each of the formulation items separately and (b) time in foster care and child age were correlated with DSED, RAD1, and RAD2. Effect sizes were interpreted using the recommendations of Cohen (1988).

## Results

The study sample (*N* = 320) were aged between 11 to 17 years (*M* = 14.5, *SD =* 2.0), they had lived in foster care for a mean of 6.6 years (*SD* = 4.3), and 56.9% were boys. Table 2 shows response frequencies of the 9 DSED items, the 11 RAD items, and their corresponding *DSM-5* criteria, as well as the 13 formulation items in the RADA.

In the DSED subscale, the item *Does she/he need to be in center of attention* was the most frequently confirmed item (*M* = 0.83, *SD* = 0.80), with 58.1% of foster parents rating this behavior as occurring “A little” or “A lot.” The two items measuring indiscriminate relationships were the second most frequently confirmed behaviors: Indiscriminate peer relationships (*M* = 0.43, *SD* = 0.70) were rated as occurring either “A little” or “A lot” by 30.3% of the foster parents. Indiscriminate relationships with adults (*M* = 0.39, *SD* = 0 .65) were confirmed by 30% of the foster parents. Regarding the RAD subscale, the item “Limited positive affect” (*M* = 0.93, *SD* = 0.84) had the highest frequency, with 45% of foster parents confirming this behavior occurring “A little” or “A lot.” “Inability to seek comfort” (*M* = 0.66, *SD* = 0.67) and “Difficulties being affectionate” (*M* = 0.66, *SD* = 0.71) were occurring “A little” or “A lot” according to 55.1% and 51.9% of the foster parents, respectively.

### Internal Validity

The hypothesized two-factor model showed a poor fit to our data (χ^2^ = 4218.066, *df* = 190, *p* < .001, CFI = 0.85, TLI = 0.83, RMSEA = 0.11, 90% confidence interval [CI] [0.10, 0.11]). In the alternative three-factor model, the RAD items were divided into two factors: RAD1 consisting of Item 10, “*Inability to seek comfort*,” and Item 11, “*Inability to accept comfort*,” comprising Criteria A; RAD2 consisting of Items 12 to 20, comprising Criteria B. The third factor consisted of the DSED items. This model showed an improved but not good fit to our data (χ^2^ = 6137.020, *df* = 190, *p* < .001, CFI = 0.91, TLI = 0.90, RMSEA = 0.10, 90% CI [0.09, 0.109]). Examination of Modification indices (MI) revealed that Item 16 (Difficulties being affectionate) in RAD2 cross-loaded with RAD1 (MI 126.79). The adjusted three-factor model accounting for Item 16 cross-loading on the RAD1 factor showed a good fit to our data (χ^2^ = 6137.020, *df* = 190, *p* < .001, CFI = 0.94, TLI = 0.95, RMSEA = 0.08, 90% CI [0.07, 0.09]). Item 16 had a loading on RAD 1 at 0.64. The chi-square test identified a significantly better fit for this three-factor model (*df* = 167, χ^2^ = 355.60) compared with the two-factor model (*df* = 169, χ^2^ = 735.72; Difftest: χ^2^ 37.995, *df* = 2, *p* < .001). Table 3 shows the factor loadings for the modified three latent factors in the RADA. The ω coefficients derived from the results of the CFA with three factors showed acceptable to high reliability for DSED (.88), RAD1 (.77), and RAD2 (.69). Correlations between the latent factors DSED and RAD1 were .08; DSED and RAD2 had a correlation of .54; and RAD1 and RAD2 had a correlation of .37.

**Table 3.** Latent Factor Loadings of DSED, RAD1, and RAD2 Items (*N* = 320).

Item no.		Factor loadings		
		F1	F2	F3
	*DSED items*			
1	Indiscriminate adult relationship	0.84		
2	Cuddliness with strangers	0.83		
3	Comfort seeking with strangers	0.48		
4	Personal questions	0.78		
5	Invading social boundaries	0.67		
6	Minimal checking back	0.50		
7	Wandering off with a stranger	0.72		
8	Indiscriminate peer relationships	0.81		
9	Demanding/attention seeking	0.58		
	*RAD items*			
10	Inability to seek comfort		0.90	
11	Inability to accept comfort		0.90	
12	Emotional and social withdrawal			0.68
13	Avoids eye contact			0.67
14	Avoids physical contact			0.57
15	Limited positive affect			0.28
16	Difficulties being affectionate			0.01
17	Emotional unpredictability			0.69
18	Approach/avoidance to carers			0.81
19	Hypervigilance			0.71
20	Frozen watchfulness			0.65

*Note.* DSED = disinhibited social engagement disorder; RAD = reactive attachment disorder.

A post hoc examination of the MI showed that the DSED Item 6, “*Minimal checking back*,” had rather large cross loadings (>.50) on both RAD1 and RAD2. Removal of this item led to good fit of the model to our data (χ^2^ = 5819.516, *df* = 171, *p* < .001, CFI = 0.96, TLI = 0.95, RMSEA = 0.07, 90% CI [0.06, 0.08]).

For the DSED subscale comprising nine items, the mean scale score was 0.37 (*SD* 0.39, range 1.78, Cronbach’s α = .80, Skewness 1.3, Kurtosis 1.1). For the RAD1 scale comprising two items, the mean scale score was 0.60 (*SD* 0.58, range 2.00, Cronbach’s α = .79, Skewness 0.5, Kurtosis −0.7). For the RAD2 scale comprising nine items, the mean scale score was 0.54 (*SD* 0.38, range 1.67, Cronbach’s α = .71, Skewness 0.6, Kurtosis −0.4).

### Relationship Between RADA Factors, Age, Time in Foster Care, and Emotional-Relational Impairment as Measured With the Formulation Items

Time in foster care was not associated with scores on DSED, RAD1, or RAD2, respectively. Higher age was associated with lower scores on DSED (*r* = −.21, *p* < .001), and higher scores on RAD1 (*r* =.26, *p* < .001).

All 12 formulation items were associated with RAD2, with Misunderstanding emotion, Need to be in control, and False affection yielding large effect size (*r* ⩾ .5). DSED was also associated with all of the formulation items but with overall lower effect sizes (*r* ⩾ .3). RAD1 showed a somewhat different pattern. Here, lack of remorse and lack of empathy showed the strongest association (*r* = .4). Results are displayed in Table 4.

**Table 4.** Correlations Between Formulation Items and the DSED, RAD1 (Failure to Seek/Accept Comfort), and RAD2 (Withdrawal/Hypervigilance) Factors.

Item no.	Formulation items	DSED, *r*	RAD1, *r*	RAD2, *r*
21	Misunderstand emotion	0.44***	0.14**	0.63***
22	Negative attitude toward self	0.26***	−0.04	0.44***
23	Self-harm	0.16*	0.08	0.29***
24	Lack of remorse	0.40***	0.45***	0.44***
25	Lack of empathy	0.15*	0.41***	0.43***
26	Need to be in control	0.39***	0.12*	0.50***
27	False affection	0.45***	0.28***	0.52***
28	Hanging on behavior	0.43***	−0.01	0.36*** *
29	Possessiveness	0.37***	0.02	0.42***
30	Pseudo-adult behavior	0.43***	−0.01	0.20*
31	Abnormal eating pattern: gorging	0.33***	−0.01	0.26***
32	Abnormal eating pattern: stealing	0.42***	0.10	0.33***

*Note. r* = Pearson’s correlation; DSED = disinhibited social engagement disorder; RAD = reactive attachment disorder.

* *p* < .05. **p < .01. ***p < .001.

## Discussion

This study is the first to modify a well-established assessment tool for RAD and DSED to correspond to the new *DSM-5* criteria and evaluate its construct validity for youth in foster care. The final version of the RADA had nine new items added, four of which were modified to better reflect the developmental stage of adolescents, by including indiscriminative behavior toward peers. Furthermore, 10 items from the original interview were removed, as they did not exclusively comply with the *DSM-5* criteria or were formulated in a way that made it hard to distinguish from more common mental health problems.

Overall, our data supported a clear distinction between the two constructs of DSED and RAD. The factor representing DSED encompasses all of the nine items measuring DSED behavior according to the *DSM-5* criteria. The factor loadings were all good to excellent, according to the criteria of Tabachnick and Fidell (2007). In line with earlier findings (Kay & Green, 2013; Lehmann et al., 2016; Minnis et al., 2013), our study shows that the dimension of DSED captures symptoms existing in maltreated children raised in a family context. However, our results also show that most of these symptoms are rather rare in this group of youth. Seventy percent or more of the parents denied that these symptoms were present in their child, with the item demanding/attention seeking being an exception. This is contrary to a previous finding among younger foster children, where DSED symptoms were more frequent than RAD symptoms (Lehmann et al., 2016). It could be that the RADA is not sensitive enough to capture the full range of DSED symptoms among older youth, or it could be that most youth in our study do not exhibit symptoms of DSED. However, our findings are in line with Humphreys et al. (2017), where RAD signs were higher than DSED signs, for both ever institutionalized and controls at the age of 12 years. Further research is needed on youth populations to conclude whether DSED symptoms decline in adolescence as a general tendency.

The DSED Item 6 (Minimal checking back, Criteria A3) showed high cross-loadings with both RAD1 and RAD2. This item had been amended to make it more appropriate for adolescents and was worded: *Some young people act too independent for his/her age, for example by failing to let you know where she/he is and when she/he is coming back. Is she/he like that?* Nearly 28% of the foster parents recognized this behavior in their youth. Still, our finding indicates that this item does not capture the behavior corresponding exclusively to DSED Criterion A3 for adolescents. Other groups of researchers have investigated indiscriminate behavior with adoptive parents of institutionalized children with use of the Five Item Indiscriminately Friendliness Behavior interview (5FI). In line with our finding, the 5FI item *Wandering off without distress* has been found to correlate weakly or not at all with other DSED items for cares of previously maltreated children (Dobrova-Krol et al., 2010; Pears et al., 2010). The same result was found with use of the ETRAD-Q in school-aged children (Monette et al., 2017). The issue could be that DSED Criterion A3 has both characteristics related to social disinhibition as well as to lack of social reference to caregivers, similar to behaviors associated with RAD. A further question therefore may be the specificity of Criterion A3 for DSED. Further studies using the RADA are needed to assess whether differently formulated items enable assessment of this criteria or whether Item 6 should be removed from the instrument.

According to our findings, the construct of RAD may be categorized into two subconstructs, in accordance with Criteria A and B in *DSM-5*. The first factor, RAD1, seems to regroup Criteria A1 and A2: *A pattern of inhibited, emotionally withdrawn behavior toward caregivers, manifested by both minimal seeking and accepting comfort when distressed*. Hence, this factor captures lack of attachment behavior. In the current version of the RADA, RAD1 comprises only two items, *Inability to seek comfort* and *Inability to accept comfort*. But we found that Item 16, *Difficulties being affectionate*, also had high loading on RAD1. As much as 52% of the foster parents in our study readily confirmed this behavior occurring a little or a lot in their foster youth. If future examination of the RADA in other samples confirms our findings, Item 16 could be part of RAD1, indicating lack of attachment behavior.

RAD1 relates to Criteria A1 and A2, suggesting that the child has no or minimal attachment to the caregiver. However, the interpretation of these results must take into account the fact that respondents are foster parents of older children/youth, with variable time spent in foster care. Consequently, these behaviors may reflect the foster child’s cautious relationship with the foster parents, rather than a lack of ability to form selective attachments as such. In their review, Zeanah and Gleason (2015) conclude that while RAD symptoms decrease with time in a nurturing foster placement, DSED symptoms seem more persistent in some children. We did not find any relation between time in foster care and scores on DSED and RAD. However, our sample represents a group of youths who are in relatively stable and long-term placements (mean duration of 6.6 years in the current foster home), and our results may be influenced by a limited variation in time spent in foster care.

The second subfactor, RAD2, comprises items intended to cover Criteria B1, 2, and 3 (withdrawal/hypervigilance). The factor loadings were all strong (⩾.5). Social neglect is a diagnostic requirement of both RAD and DSED. For maltreated children growing up in severely troubled families before placement, emotional neglect and fear-provoking behavior in carers often go together. The experience for the child might include exposures contributing to both RAD2 symptoms and DSED symptoms. However, it is worth noticing the differential correlation between RAD and DSED depending on RAD subfactors. While the correlation between DSED and RAD1 was near 0, DSED and RAD2 had a correlation of .55. This strengthens the notion of RAD1 and RAD2 as distinct and separate constructs. One might speculate that while RAD1 seems to capture more pure attachment-related difficulties, items comprising RAD2 are more closely related to relational trauma caused by maltreatment, and hence may occur alongside both difficulties in establishing selective attachment (RAD1) and social aberrant behavior (DSED).

The most striking finding from our correlation matrix of formulation items with the RAD1, RAD2, and DSED factors was the low associations between the formulation items and the RAD1 factor relative to DSED and RAD2. Only Lack of remorse and Lack of empathy were moderately associated with RAD1. It could be hypothesized that RAD1 represents a behavior that stands out as rather unrelated to other more common clinical symptoms. Our results strengthen the notion of RAD1 representing a purer measure of lack of selective attachment. The finding that the callous and unemotional (CU) items Lack of empathy and Lack of remorse were associated with both RAD1 and RAD2 is worth noticing. Mayes, Calhoun, Waschbusch, Breaux, and Baweja (2017) found that RAD seems to be more associated with CU traits than DSED in maltreated children in foster care. Severe early deprivation (Humphreys et al., 2015), as well as poor positive parenting in low-income families (Waller, Shaw, & Hyde, 2017), seem to increase the risk for CU traits. These risk factors are often present in the foster care population, and attachment-related difficulties may be the common outcome of both deprivation and negative parenting styles. As CU traits in childhood have been linked to adult psychopathy (Frick, Ray, Thornton, & Kahn, 2014) a possible overlap between severe early neglect, attachment disorders, and later developmental/emergent psychopathic tendencies needs to be examined further in longitudinal studies. It is also pertinent that RAD2 was associated with all 12 formulation items. This finding strengthens our interpretation of RAD2 as related to relational trauma with broad consequences for the child’s mental health and interpersonal functioning.

### Strengths and Limitations

The key strength of this study is the examination of RAD and DSED traits in older youth based on *DSM-5*. Also, the study was a collaborative effort of an international team of researchers, conducting a review of existing assessment tools and a thorough revision of an established assessment tool to ensure correspondence with changes in the *DSM-5*. Thus, this study is the first to examine RAD and DSED behavior in older youths within the *DSM-5* framework. Furthermore, the study included a large sample that is representative of youth in foster homes. Of the total sample of 405 foster youth, nearly 80% (320) foster parents completed the RADA; yet despite high completion rate, the 20% attrition raises a risk of nonresponse bias. The focus on a Norwegian sample also decreases the generalizability of our results.

Furthermore, the ambiguous role of Item 6 (Minimal checking back) with an adequate loading (.44) on the DSED factor and a substantial cross-loading to the RAD1 (.55) and the RAD2 (.51) factors indicates a substantial problem with this item in identifying children with DSED. Further examination of the appropriateness of this item in measuring DSED behavior is needed in studies with different samples and age range. In contrast, if further studies replicate the finding that RAD consists of two subfactors, the use of formulation items together with Item 16 (Difficulties being affectionate) to increase the number of items in this factor should be considered.

As the empirical foundation for the construct of RAD and DSED behavior in adolescents is minimal, future studies on different age-groups and risk profiles are needed to examine the discriminant ability and relevance of the formulation items for the RAD and DSED dimensions (Minnis et al., 2002). In addition, the factor structure and loadings found in this study needs to be further examined in large-scale studies.

### Use of the RADA in Research and Clinical Settings

Both the semistructured RADA interview and the online version allow for measurement of RAD and DSED behavior as dimensional constructs in both clinical and research settings. A dimensional approach provides valuable information on child needs and functioning, especially when used together with measures of other, more common mental health problems. For diagnostic purposes, RADA may be used to generate research diagnoses in larger epidemiological studies, ideally alongside reports from other informants such as teachers and via structured observation to provide a multi-informant diagnosis. The online version has a clear advantage for this use, as it enables completion from informants with low administration resources.

In clinical practice, following the practice recommendations from Zeanah et al. (2016), screening-tools such as RPQ or ETRAD-Q may be used as a first step. High scorers should then be offered further assessment with use of the RADA interview alongside the teacher Relationship Problem Questionnaire (Minnis et al., 2002) and observational measures such as the waiting room observation procedure (McLaughlin et al., 2010), which explore the interaction between the child and stranger(s) on first meeting (Minnis et al., 2013).
